# Psychiatrists, public mental health and sustainability: understanding the overlap and taking action

**DOI:** 10.1192/bjb.2025.10140

**Published:** 2026-06

**Authors:** Natalie Cook, Katie Blissard Barnes

**Affiliations:** 1 Tees, Esk and Wear Valley NHS Foundation Trust, Darlington, UK; and RCPsych Planetary Health and Sustainability Committee, London, UK; 2 Leeds and York Partnership NHS Foundation Trust, Leeds, UK; and RCPsych Planetary Health and Sustainability Committee, London, UK

**Keywords:** Public mental health, sustainability, psychiatrists

## Abstract

**Aims and method:**

Through examining three areas of focus within public mental health; prevention, healthy environments and reducing inequalities, we consider how interventions in these domains also have the potential to create a more sustainable healthcare system.

**Results:**

We show how psychiatrists and mental health professionals alongside the wider health and social care system can be involved in advocating for this change.

**Clinical implications:**

We aim to empower individuals working within mental health to advocate for change and consider how public mental health approaches can be integrated into their practice to improve outcomes.

Public mental health is a population-based approach to improving mental well-being in society through evidence-based interventions that prevent mental ill-health and promote recovery.^1,2^ It requires multisectoral working across health and social care, education and the workplace, with valuable contributions from the voluntary sector. If we consider the triple bottom line approach, which measures a business’s success by its social, environmental and economic benefits, we should be trying to provide the most effective interventions to prevent mental illness in a cost-effective way that has the least impact on the environment.^3^ This is particularly important when we consider that the National Health Service (NHS) currently contributes around 4–5% of total UK carbon emissions, with a goal of becoming net zero by 2040.^4^

Despite the existence of evidence-based public mental health interventions, there is an implementation gap, meaning that few are offered these interventions. This gap is even more evident in low- and middle-income countries.^5^ This health inequality is exacerbated further by the ‘inverse climate law’, where those at greatest risk from the climate crisis have the least resilience and available resources.^6^ The 2024 Europe report of the Lancet Countdown highlights the links between health and climate change, focusing on the inequalities within countries and across the continent.^7^ Many of the climate-related indicators mentioned in the report have an impact on mental health – for example, heatwaves, food insecurity, availability of green spaces, air pollution and diet.^7^

In this article, we explore how addressing these challenges not only promotes public mental health but also contributes to building more sustainable healthcare systems. We focus on the prevention of mental illness, healthy environments and reducing health inequalities as they represent key points of intersection between sustainability and public mental health, offering opportunities for social, environmental and economic improvements. These areas have also been highlighted as key commitments for the current government, alongside creating a sustainable NHS.^8^

We will demonstrate how we can simultaneously improve mental health outcomes and ensure healthcare systems are better equipped to adapt to and mitigate the impacts of climate change. We also want to empower individuals working within mental health to make changes to their own practice as well as advocate for wider trust-level or NHS-wide changes.

## Prevention of mental illness

Many factors affect mental health and, if addressed, may prevent mental ill-health at an individual and population level. Primary prevention is a core focus of public mental health interventions, with the aim of stopping people from developing mental health difficulties through a range of approaches that benefit the whole community. This may include parenting programmes and promoting parent–child attachment;^9,10^ alcohol, drug and smoking reduction campaigns;^11,12^ encouraging physical activity;^13,14^ teaching emotional learning in schools;^15^ optimising workplace well-being;^16^ and anti-stigma campaigns.^17^

Secondary prevention is focused on the early identification of illness, particularly in individuals who are at higher risk, which may be related to life events, protected characteristics (attributes covered by equality law, such as race, religion and gender), migration or those with trauma histories among many other factors. Interventions may include early identification of psychosis through Early Intervention in Psychosis teams,^18^ screening for postnatal depression or the provision of targeted support for at-risk groups such as migrants.^19^

Finally, tertiary prevention aims to manage symptoms for those with an established mental health condition to provide the best chance of recovery, prevent relapse and maximise quality of life. Interventions may include improving the physical health of those with severe mental illness^20^ and return-to-work support through Individual Placement and Support workers integrated in mental health teams.^21^ There is, of course, overlap between these areas of prevention, and not all interventions fit neatly into the three categories.

Prevention is a cornerstone of both sustainable healthcare and public mental health. For psychiatrists, preventing the onset or reducing the impact of mental illness could result in less demand on mental health services, which would reduce waiting lists and caseload numbers. Earlier intervention could mean more care in the community, easing pressure on bed numbers, while offering a more affordable and less carbon-intensive model of care.^22^ Many interventions offered with prevention in mind also align with the recovery model, a personalised holistic model of care encouraged within the NHS.^23^

Preventative interventions often lead to personal, societal and environmental gains. For instance, research shows that nearly half of all dementia cases could be prevented by tackling 14 risk factors, which include smoking, obesity, inactivity, air pollution and social isolation.^24^ Population-level interventions such as clean air policies and measures to reduce salt intake and reduce smoking will not only improve quality of life, but also save up to £4 billion in England alone.^24^

## Reducing health inequalities

The National Institute for Health and Care Excellence defines health inequalities as ‘differences in health across the population, and between different groups in society, that are systematic, unfair and avoidable’.^25^ These disparities are experienced by many, including those living in poverty, those belonging to societal groups more at risk of social exclusion (e.g. migrants and refugees) and individuals with certain protected characteristics.^26^ When a person belongs to multiple marginalised groups, such as being a refugee and identifying as LGBTQIA+, this intersectionality amplifies the impact.^27^

As psychiatrists, we frequently encounter the disproportionate mental health burden borne by vulnerable groups. Poverty, for instance, both contributes to and may be exacerbated by mental illness.^28^ Migrants and refugees face significant challenges such as discrimination, poor working conditions and financial insecurity, which may elevate their risk of developing mental illness or exacerbate pre-existing conditions, while simultaneously creating barriers to accessing care.^29^ While there is a push to explore the role of artificial intelligence and digital solutions in healthcare, certain populations are more vulnerable to digital poverty, thus reducing their access to this form of support and widening these inequalities even further.^30^

Tackling these inequalities requires both targeted and systemic action. Improving access to services, embedding cultural competence and tailoring care to specific communities are essential for reducing disparities. Equally, mental health professionals must be supported to address the broader social determinants of health – such as housing, education and financial security – that underpin many of the inequities we observe in clinical practice.

Health inequalities also intersect with sustainability challenges. Climate change represents a health inequality because the impacts – such as extreme weather, food insecurity and displacement – disproportionately affect the most disadvantaged communities, who have the fewest resources to adapt and recover. These communities, as described above, often already experience poorer physical and mental health outcomes, and climate-related stressors further exacerbate existing health disparities by increasing exposure to trauma, instability and environmental hazards.

The Global South, for example, is disproportionately affected by climate change, with greater exposure to extreme weather events, subsequent displacement and risk of food insecurity – all of which increase the risk of forced migration and consequent mental health risks.^31,32^ Not only are those most disadvantaged in society at most risk from the consequences of climate change, but as the impacts of climate change increase, so it will exacerbate and widen these disparities.^6^ This represents a significant global and national challenge. Within the UK, for example, those living with higher levels of deprivation are also more likely to live in flood-prone areas, have poorly insulated housing and experience high levels of air pollution.^33–35^ These same individuals are at greater risk of mental illness, perpetuating a cycle of disadvantage. Environmental hazards, such as air and noise pollution, often prevalent in deprived neighbourhoods, also contribute to worse mental health outcomes.^36–38^ Poor quality housing can directly impact mental health as well as increase the potential consequences from climate events such as heat waves and flooding, with issues like damp, mould and overcrowding.^33–35,39^

## Healthy environments

Our local environment plays a vital role in both public mental health and sustainability, making it a critical area of focus for psychiatrists. When we refer to environments, this may include the built environment (i.e. those areas that have been physically built) and the natural environment, which includes green spaces such as parks, nature reserves and woodland, as well as blue spaces such as rivers, seas and lakes. All of these elements can positively or negatively impact mental well-being, as well as either exacerbate climate challenges or support adaptive responses to climate change. Access to nature has profound effects on mental well-being. Research demonstrates that people living in neighbourhoods with more green space experience lower rates of anxiety and depression and report better overall well-being.^40,41^ Time spent in natural environments not only supports primary prevention of mental illness but also leads to meaningful improvements in conditions such as depression, anxiety and stress.^42^ Nature also provides opportunities for physical activity, social connection and engaging in hobbies, further enhancing mental health.

A healthy environment promotes benefits that extend beyond individual health. Green spaces play a key role in climate change adaptation by providing shade, cooling urban heat islands and reducing flood risks.^43,44^ This will become increasingly important in a world afflicted by warming temperatures. These spaces also improve local biodiversity, helping ecosystems adapt to the challenges of a changing climate. Access to these resources, however, is not evenly distributed.

Disparities in access to nature highlights the intersection between environmental and mental health disparities.^45^ People living in the most deprived areas of the UK have the least access to green spaces and are more vulnerable to climate impacts like heatwaves and flooding.^45,46^ While there is often poorer access to protective natural environments that enhance resilience, so there is also a higher exposure to environmental factors that impact mental health.

The environments we live in are also heavily influenced by commercial determinants of health, contributing to inequalities and impacting public mental health: ‘Commercial determinants of mental health describe how actions of private companies affect people’s mental health.’^47,48^ Once again, vulnerable groups are disproportionately targeted and impacted, for example, fast-food outlets and alcohol retailers are more prevalent in deprived neighbourhoods, contributing to higher rates of obesity, addiction and related mental health conditions in these areas.^49,50^ Factories emitting high levels of air pollution are also more likely to be found in deprived communities.^51^ These same commercial interests drive a high-consumption society that is inherently unsustainable, while also contributing to poor mental health outcomes. This stands in contrast to the goals of a truly sustainable healthcare system, in which prevention is prioritised, equity is the norm and environmental impacts are minimised.

## Implications for practice

Psychiatrists have a unique opportunity to support prevention, address health inequalities and contribute to a more sustainable mental healthcare system. By adopting a holistic and proactive approach, they can drive meaningful change at the individual, service and system levels.

### Individual level

- Incorporate prevention and environmental factors into routine assessments, including questions about smoking, physical activity, social support, housing quality, exposure to pollution and access to green and blue spaces
- Proactively identify individuals at higher risk of developing mental illness – such as those who have experienced trauma, belong to minoritised groups or present with early signs of illness – and offer timely assessments or referrals
- Collaborate with multidisciplinary teams to promote social recovery and mental well-being by signposting patients to community resources such as:
  1. Smoking cessation or weight management programmes
  2. Parenting classes or workplace well-being schemes
  3. Support groups – for example, Andy’s Man Club and Age UK
  4. Nature-based and social prescribing activities – for example, gardening groups, conservation volunteering and walking groups^52,53^
- Support integrated physical and mental healthcare, including advocating for metabolic monitoring and health checks in patients with severe mental illness
- Address commercial and social determinants of mental health by exploring the impact of poor diet, substance use or local environmental stressors on mental well-being
- Encourage use of relapse prevention plans and crisis contingency planning in routine care, including co-production with patients and carers
- Identify environmental and social contributors to relapse, such as housing instability or social isolation, and work with community services to mitigate them

### Service level

- Advocate for inclusive, person-centred services aligned with the NHS Long Term Plan^54^ – delivering care closer to home and tailored to the needs of diverse populations
- Promote equitable access to care by identifying and addressing barriers faced by marginalised populations, including language, cultural, financial and digital access challenges
- Engage with underserved communities through outreach, peer support and partnerships with voluntary and community sector organisations
- Encourage team awareness and training on how social and environmental contexts – such as housing quality and access to green space – impact mental health, especially in communities experiencing high deprivation; and encourage incorporating these considerations into clinical assessments and care planning
- Embed cultural competence and trauma-informed approaches into service design and clinical practice, including training for all mental health staff
- Advocate for and contribute to greener mental health facilities, including:
  1. Creating or enhancing access to natural spaces on in-patient sites (e.g. tree planting)
  2. Incorporating nature into out-patient environments
  3. Developing therapeutic horticulture and ecotherapy programmes within occupational therapy or recovery services, particularly in in-patient or long-stay settings
  4. Partnering with local charities – one example of this is the partnership between Foss Park Hospital in York and St Nicks who are working to develop the green space on the hospital site and provide gardening opportunities to patients through occupational therapy^55^

### System level

- Champion upstream and preventative policies by collaborating with public health teams, local authorities and other sectors – for example, housing and transport
- Provide expert input on mental health impacts of sustainable infrastructure, including clean air zones, active travel schemes and green space access
- Advocate for environmental justice and equitable access to protective environments, particularly in marginalised or climate-vulnerable communities
- Challenge commercial drivers of mental ill-health, such as the clustering of fast-food, alcohol or gambling outlets in deprived areas
- Highlight the links between sustainability and mental health in service planning and professional forums
- Support policy change at local and national levels that addresses the social determinants of mental health and aim to mitigate climate-related risks for vulnerable populations
- Engage with your Trust’s Green Plan by educating colleagues about its goals and actively implementing sustainable practices within your clinical team or service

### Understanding the overlap

Public mental health and sustainability are deeply interconnected, with shared goals of improving well-being, reducing inequalities and delivering the right care at the right time in the right place. By framing healthy environments, the prevention of mental illness and health inequalities through a sustainability lens, we can identify interventions that not only promote mental well-being but also address environmental challenges.

For psychiatrists, this overlap represents both an opportunity and a responsibility. Individual actions, such as incorporating environmental factors into patient assessments or promoting access to green spaces, can have a meaningful impact. At the same time, systemic advocacy for sustainable healthcare practices and equitable policies is essential for creating lasting change.

An understanding of the principles of sustainability and how they underpin sustainable psychiatric practice is now incorporated into the curriculum.^56^ Consequently, we would like to see further support to equip psychiatrists with the knowledge and skills to address these challenges effectively, through offerings such as the Public Mental Health Leadership course available through the Public Mental Health Implementation Centre.^57^

The links between public mental health and sustainability become even clearer when considering the core principles of practising sustainably, as described by the Royal College of Psychiatry Planetary Health and Sustainability Committee:^58^

1. Prioritising prevention
2. Empowerment of patients, communities and staff
3. High value care
4. Consider carbon

Alongside the committee, the College have also produced several eLearning modules with a focus on topics key to sustainability, which can be accessed via the College website.^59^ Addressing the gaps in awareness will be crucial to advancing this agenda. Equipping mental health professionals with the knowledge to act on these issues and generating reliable and robust data on the impacts of sustainable public mental health strategies will ensure interventions are both effective and equitable.

Ultimately, by adopting an approach that integrates public mental health and sustainability, we can contribute to a healthcare system that promotes mental health while protecting the planet for future generations – a truly sustainable future.

## Data Availability

Data availability is not applicable to this article as no new data were created or analysed in this study.

## About the authors

**Natalie Cook** is a consultant psychiatrist at Tees, Esk and Wear Valley NHS Foundation Trust and member of the RCPsych Planetary Health and Sustainability Committee. **Katie Blissard Barnes** is a ST7 psychiatrist at Leeds and York Partnership NHS Foundation Trust and member of the RCPsych Planetary Health and Sustainability Committee.

## References

[1] Royal College of Psychiatrists (RCPsych). *Public Mental Health Implementation Centre* . RCPsych, n.d. (https://www.rcpsych.ac.uk/improving-care/public-mental-health-implementation-centre [accessed 12 Mar 2025]).

[2] Faculty of Public Health, Mental Health Foundation. *Better Mental Health for All: A Public Health Approach to Mental Health Improvement*. UK Faculty of Public Health, 2016 (https://www.mentalhealth.org.uk/sites/default/files/2022-09/MHF-better-mental-health-for-all.pdf [accessed 12 Mar 2025]).

[3] Centre for Sustainable Healthcare. *Sustainable Quality Improvement (SusQI)*. Centre for Sustainable Healthcare, n.d. (https://sustainablehealthcare.org.uk/sustainable-quality-improvement-susqi/ [accessed 12 Mar 2025]).

[4] NHS England. *Delivering a ‘Net Zero’ National Health Service*. NHS England, 2022 (https://www.england.nhs.uk/greenernhs/wp-content/uploads/sites/51/2022/07/B1728-delivering-a-net-zero-nhs-july-2022.pdf [accessed 12 Mar 2025]).

[5] Campion J. Public mental health: key challenges and opportunities. BJPsych Int 2018; 15: 51–4. 31452534 10.1192/bji.2017.11 PMC6690256

[6] Bede F, Radcliffe E. *The ‘Inverse Climate Law’: A Call for Health Equity and Climate Justice*. Bjgplife.com, 2022 (https://bjgplife.com/the-inverse-climate-law-a-call-for-health-equity-and-climate-justice/ [accessed 12 Mar 2025]).

[7] van Daalen KR , Tonne C , Semenza JC , Rocklöv J , Markandya A , Dasandi N , et al. The 2024 Europe report of the Lancet Countdown on health and climate change: unprecedented warming demands unprecedented action. Lancet Public Health 2024; 9: e495–522. 38749451 10.1016/S2468-2667(24)00055-0 PMC11209670

[8] Davie E. *A Mentally Healthier Nation*. Centre for Mental Health, 2023 (https://www.centreformentalhealth.org.uk/publications/mentally-healthier-nation/ [accessed 12 Mar 2025]).

[9] Costantini I , López-López JA , Caldwell D , Campbell A , Hadjipanayi V , Cantrell SJ , et al. Early parenting interventions to prevent internalising problems in children and adolescents: a global systematic review and network meta-analysis. BMJ Mental Health 2023; 26: e300811. 10.1136/bmjment-2023-300811 PMC10619111 37907332

[10] Al Sager A , Goodman SH , Jeong J , Bain PA , Ahun MN. Effects of multi-component parenting and parental mental health interventions on early childhood development and parent outcomes: a systematic review and meta-analysis. Lancet Child Adolesc Health 2024; 8: 656–69. 39142740 10.1016/S2352-4642(24)00134-2

[11] National Institute for Health and Care Excellence (NICE). *Tobacco: Preventing Uptake, Promoting Quitting and Treating Dependence*. NICE, 2025 (https://www.nice.org.uk/guidance/ng209 [accessed 12 Mar 2025]). 36745727

[12] Das JK , Salam RA , Arshad A , Finkelstein Y , Bhutta ZA. Interventions for adolescent substance abuse: an overview of systematic reviews. J Adolesc Health 2019; 59: S61–75. 10.1016/j.jadohealth.2016.06.021 PMC5026681 27664597

[13] Hu MX , Turner D , Generaal E , Bos D , Ikram MK , Ikram MA , et al. Exercise interventions for the prevention of depression: a systematic review of meta-analyses. BMC Public Health 2020; 20: 1255. 32811468 10.1186/s12889-020-09323-y PMC7436997

[14] Mueller N , Rojas-Rueda D , Cole-Hunter T , de Nazelle A , Dons E , Gerike R , et al. Health impact assessment of active transportation: a systematic review. Prev Med 2015; 76: 103–14. 25900805 10.1016/j.ypmed.2015.04.010

[15] Murano D , Sawyer JE , Lipnevich AA. A meta-analytic review of preschool social and emotional learning interventions. Rev Educ Res 2020; 90: 227–63.

[16] National Institute for Health and Care Excellence (NICE). *Mental Wellbeing at Work*. NICE, 2022 (https://www.nice.org.uk/guidance/ng212 [accessed 12 Mar 2025]). 35471805

[17] Time to Change. *A Campaign to Change the Way People Think and Act about Mental Health Problems*. Time to Change, n.d. (https://www.time-to-change.org.uk/ [accessed 12 Mar 2025]).

[18] Kendall T. *Implementing the Early Intervention in Psychosis Access and Waiting Time Standard*. NHS England, 2023 (https://www.england.nhs.uk/wp-content/uploads/2023/03/B1954-implementing-the-early-intervention-in-psychosis-access-and-waiting-time-standard.pdf [accessed 12 Mar 2025]).

[19] Stevenson K , Fellmeth G , Edwards S , Calvert C , Bennett P , Campbell OMR. The global burden of perinatal common mental health disorders and substance use among migrant women: a systematic review and meta-analysis. Lancet Public Health 2023; 8: E203–16. 36841561 10.1016/S2468-2667(22)00342-5

[20] NHS England. *Improving the Physical Health of People Living with Severe Mental Illness*. NHS England, 2025 (https://www.england.nhs.uk/long-read/improving-the-physical-health-of-people-living-with-severe-mental-illness [accessed 12 Mar 2025]).

[21] NHS England. *Individual Placement and Support Offers Route to Employment for People with Severe Mental Health Conditions*. NHS England, n.d. (https://www.england.nhs.uk/mental-health/case-studies/severe-mental-illness-smi-case-studies/individual-placement-and-support-offers-route-to-employment-for-people-with-severe-mental-health-conditions [accessed 12 Mar 2025]).

[22] Gilburt H , Mallorie S. *Mental Health 360: Acute Mental Health Care for Adults*. The King’s Fund, 2024 (https://www.kingsfund.org.uk/insight-and-analysis/long-reads/mental-health-360-acute-mental-health-care-adults [accessed 12 Mar 2024]).

[23] Rethink Mental Illness. *Recovery and Mental Illness*. Rethink Mental Illness, 2022 (https://www.rethink.org/advice-and-information/living-with-mental-illness/treatment-and-support/recovery-and-mental-illness/ [accessed 12 Mar 2025]).

[24] Livingston G , Huntley J , Liu KY , Costafreda SG, Selbæk G, Alladi S, et al. Dementia prevention, intervention, and care: 2024 report of the Lancet standing Commission. Lancet 2024; 404: 572–628. 39096926 10.1016/S0140-6736(24)01296-0

[25] National Institute for Health and Care Excellence (NICE). *NICE and Health Inequalities*. NICE, n.d. (https://www.nice.org.uk/about/what-we-do/nice-and-health-inequalities [accessed 12 Mar 2025]).

[26] The King’s Fund. *Health Inequalities in a Nutshell*. The King’s Fund, 2024 (https://www.kingsfund.org.uk/insight-and-analysis/data-and-charts/health-inequalities-nutshell [accessed 12 Mar 2025]).

[27] Tinner L , Alonso Curbelo A. Intersectional discrimination and mental health inequalities: a qualitative study of young women’s experiences in Scotland. Int J Equity Health 2024; 23: 45. 38424534 10.1186/s12939-024-02133-3 PMC10903064

[28] Elliott I. Poverty and Mental Health: *A Review to Inform the Joseph Rowntree Foundation’s Anti-Poverty Strategy*. Mental Health Foundation, 2016 (https://www.mentalhealth.org.uk/sites/default/files/2022-08/poverty-and-mental-health-report.pdf [accessed 12 Mar 2025]).

[29] Mental Health Foundation. *The Mental Health of Asylum Seekers and Refugees in the UK*. Mental Health Foundation, 2024 (https://www.mentalhealth.org.uk/sites/default/files/2024-02/MHF_Mental-Health-of-Asylum-Seekers_REPORT_A4_SINGLE-PAGES_0.pdf [accessed 12 Mar 2025]).

[30] Ibrahim H , Liu X , Zariffa N , Morris AD , Denniston AK. Health data poverty: an assailable barrier to equitable digital health care. Lancet Digital Health 2021; 3: e260–5. 33678589 10.1016/S2589-7500(20)30317-4

[31] Zhang S , Braithwaite I , Bhavsar V , Das-Munshi J . Unequal effects of climate change and pre-existing inequalities on the mental health of global populations. BJPsych Bull 2021; 45: 1–5. 33759737 10.1192/bjb.2021.26 PMC8499621

[32] Lawrance EL , Thompson R , Newberry Le Vay J , Page L , Jennings N. The impact of climate change on mental health and emotional wellbeing: a narrative review of current evidence, and its implications. Int Rev Psychiatry 2022; 34: 443–98. 36165756 10.1080/09540261.2022.2128725

[33] Hall M , Bailey P. *Social Deprivation and the Likelihood of Flooding*. Environment Agency, 2022 (https://assets.publishing.service.gov.uk/media/6270fe448fa8f57a3cdbbeb9/Social_deprivation_and_the_likelihood_of_flooding_-_report_2.1.pdf [accessed 12 Mar 2025]).

[34] The Health Foundation. *Inequalities in Likelihood of Living in Polluted Neighbourhoods*. The Health Foundation, 2024 (https://www.health.org.uk/evidence-hub/our-surroundings/air-pollution/inequalities-in-likelihood-of-living-in-polluted [accessed 12 Mar 2025]).

[35] The Health Foundation. *Inequalities between Groups of People Living in Non-decent Homes*. The Health Foundation, 2024 (https://www.health.org.uk/evidence-hub/housing/housing-quality/inequalities-between-groups-of-people-living-in-non-decent [accessed 12 Mar 2025]).

[36] Bhui K , Newbury JB , Latham RM , Ucci M , Nasir ZA , Turner B , et al. Air quality and mental health: evidence, challenges and future directions. BJPsych Open 2023; 9: e120. 37403494 10.1192/bjo.2023.507 PMC10375903

[37] Nobile F , Forastiere A , Michelozzi P , Forastiere F , Stafoggia M. Long-term exposure to air pollution and incidence of mental disorders. A large longitudinal cohort study of adults within an urban area. Environ Int 2023; 181: 108302. 37944432 10.1016/j.envint.2023.108302

[38] Newbury JB , Stewart R , Fisher HL , Beevers S , Dajnak D , Broadbent M , et al. Association between air pollution exposure and mental health service use among individuals with first presentations of psychotic and mood disorders: retrospective cohort study. Br J Psychiatry 2021; 219: 678–85. 35048872 10.1192/bjp.2021.119 PMC8636613

[39] Shelter. *The Impact of Housing Problems on Mental Health*. Shelter, 2017 (https://england.shelter.org.uk/professional_resources/policy_and_research/policy_library/research_the_impact_of_housing_problems_on_mental_health [accessed 12 Mar 2025]).

[40] Barton J , Rogerson M. The importance of greenspace for mental health. BJPsych Int 2017; 14: 79–81. 10.1192/s2056474000002051 PMC5663018 29093955

[41] Alcock I , White MP , Wheeler BW , Fleming LE , Depledge MH. Longitudinal effects on mental health of moving to greener and less green urban areas. Environ Sci Technol 2014; 48: 1247–55. 24320055 10.1021/es403688w

[42] Mental Health Foundation. *Nature: How Connecting with Nature Benefits Our Mental Health*. Mental Health Foundation, 2021 (https://www.mentalhealth.org.uk/sites/default/files/2022-06/MHAW21-Nature-research-report.pdf [accessed 12 Mar 2025]).

[43] Yu Z , Xu S , Zhang Y , Jørgensen G , Vejre H. Strong contributions of local background climate to the cooling effect of urban green vegetation. Sci Rep 2018; 8: 6798. 29717184 10.1038/s41598-018-25296-w PMC5931616

[44] Kirschner V , Macků K , Moravec D , Maňas J. Measuring the relationships between various urban green spaces and local climate zones. Sci Rep 2023; 13: 9799. 37328548 10.1038/s41598-023-36850-6 PMC10275979

[45] The Health Foundation. *Inequalities in Access to Green Space*. The Health Foundation, 2024 (https://www.health.org.uk/evidence-hub/our-surroundings/green-space/inequalities-in-access-to-green-space [accessed 12 Mar 2025]).

[46] Turner G , Israelsson J , Reynolds N , Umeh E , O’Connor L , Loveless B. Addressing access to greenspace and health inequalities in England: policy content analysis and systematic literature review. Lancet 2024; 404: S42.

[47] Davie E, Shafan-Azhar Z, Action on Smoking and Health. *The Commercial Determinants of Mental Health*. Centre for Mental Health, 2025 (https://www.centreformentalhealth.org.uk/publications/the-commercial-determinants-of-mental-health/ [accessed 12 Mar 2025]).

[48] Dun-Campbell K , Hartwell G , Maani N , Tompson A , van Schalkwyk MC , Petticrew M. Commercial determinants of mental ill health: an umbrella review. PLOS Global Public Health 2024; 4: e0003605. 39196874 10.1371/journal.pgph.0003605 PMC11355563

[49] The Health Foundation. *Inequalities in Concentration of Fast Food Outlets*. The Health Foundation, 2024 (https://www.health.org.uk/evidence-hub/our-surroundings/access-to-amenities/inequalities-in-concentration-of-fast-food [accessed 12 Mar 2025]).

[50] Shortt NK , Tisch C , Pearce J , Mitchell R , Richardson EA , Hill S , et al. A cross-sectional analysis of the relationship between tobacco and alcohol outlet density and neighbourhood deprivation. BMC Public Health 2015; 15: 1014. 26437967 10.1186/s12889-015-2321-1 PMC4595054

[51] Walker G , Bickerstaff K. *Polluting the Poor: An Emerging Environmental Justice Agenda for the UK?* Goldsmiths College, 2000 (https://research.gold.ac.uk/id/eprint/33837/7/31.%20Gordon%20Walker%20and%20Karen%20Bickerstaff.pdf [accessed 12 Mar 2025]).

[52] NHS England. *Green Social Prescribing*. NHS England, n.d. (https://www.england.nhs.uk/personalisedcare/social-prescribing/green-social-prescribing/ [accessed 12 Mar 2025]).

[53] Royal College of Psychiatrists (RCPsych), Royal College of Occupational Therapists, Muir Wood M, NHS England. *Social Prescribing*. RCPsych, 2021 (https://www.rcpsych.ac.uk/docs/default-source/improving-care/better-mh-policy/position-statements/position-statement-ps01-21---social-prescribing---2021.pdf?sfvrsn=2b240ce4_2 [accessed 12 Mar 2025]).

[54] NHS. *The NHS Long Term Plan*. NHS, 2019 (https://www.england.nhs.uk/wp-content/uploads/2022/07/nhs-long-term-plan-version-1.2.pdf [accessed 12 Mar 2025]).

[55] Royal College of Psychiatrists (RCPsych). *2022 Curricula Implementation Hub*. RCPsych, n.d. (https://www.rcpsych.ac.uk/training/curricula-and-guidance/curricula-implementation [accessed 12 Mar 2025]).

[56] Royal College of Psychiatrists (RCPsych). *The Public Mental Health Leadership Certification Course*. RCPsych, n.d. (https://www.rcpsych.ac.uk/events/conferences/rcpsych-certificated-courses/public-mental-health-leadership-certification-course [accessed 12 Mar 2025]).

[57] Royal College of Psychiatrists (RCPsych). *Sustainability at RCPsych*. RCPsych, n.d. (https://www.rcpsych.ac.uk/improving-care/sustainability-and-mental-health/sustainability-at-rcpsych [accessed 12 Mar 2025]).

[58] Monsell A , Krzanowski J , Page L , Cuthbert S , Harvey G. What mental health professionals and organisations should do to address climate change. BJPsych Bull 2021; 45: 215–21. 33947498 10.1192/bjb.2021.17 PMC8499631

[59] Royal College of Psychiatrists (RCPsych). *CPD eLearning*. RCPsych, n.d. (https://elearninghub.rcpsych.ac.uk/catalog?pagename=CPD-eLearning [accessed 12 Mar 2025]).

